# A qualitative study on overdose response in the era of COVID-19 and beyond: how to spot someone so they never have to use alone

**DOI:** 10.1186/s12954-021-00530-3

**Published:** 2021-08-05

**Authors:** Melissa Perri, Natalie Kaminski, Matthew Bonn, Gillian Kolla, Adrian Guta, Ahmed M. Bayoumi, Laurel Challacombe, Marilou Gagnon, Natasha Touesnard, Patrick McDougall, Carol Strike

**Affiliations:** 1 Dalla Lana School of Public Health, University of Toronto, Toronto, ON Canada; 2 MAP Center for Urban Health Solutions, Li Ka Shing Knowledge Institute, St. Michael’s Hospital, Unity Health Toronto, Toronto, ON Canada; 3 Canadian Association of People Who Use Drugs, Dartmouth, NS Canada; 4 Canadian Students for Sensible Drug Policy, Toronto, Canada; 5 Canadian Institute for Substance Use Research, University of Victoria, Victoria, British Columbia Canada; 6 School of Social Work, University of Windsor, Windsor, ON Canada; 7 Institute of Health Policy, Management and Evaluation, University of Toronto, Toronto, ON Canada; 8 CATIE, Toronto, ON Canada; 9 Dr. Peter AIDS Foundation, Vancouver, BC Canada; 10 Division of General Internal Medicine, Department of Medicine, St. Michael’s Hospital, Unity Health Toronto, Toronto, ON Canada; 11 Department of Medicine, University of Toronto, Toronto, ON Canada

**Keywords:** Spotting, Overdose prevention, Digital, COVID-19, Harm reduction, Digital health interventions

## Abstract

**Background:**

Spotting is an informal practice among people who use drugs (PWUD) where they witness other people using drugs and respond if an overdose occurs. During COVID-19 restrictions, remote spotting (e.g., using a telephone, video call, and/or a social media app) emerged to address physical distancing requirements and reduced access to harm reduction and/or sexually transmitted blood borne infection (STBBI’s) prevention services. We explored spotting implementation issues from the perspectives of spotters and spottees.

**Methods:**

Research assistants with lived/living expertise of drug use used personal networks and word of mouth to recruit PWUD from Ontario and Nova Scotia who provided or used informal spotting. All participants completed a semi-structured, audio-recorded telephone interview about spotting service design, benefits, challenges, and recommendations. Recordings were transcribed and thematic analysis was used.

**Results:**

We interviewed 20 individuals between 08/2020–11/2020 who were involved in informal spotting. Spotting was provided on various platforms (e.g., telephone, video calls, and through texts) and locations (e.g. home, car), offered connection and community support, and addressed barriers to the use of supervised consumption sites (e.g., location, stigma, confidentiality, safety, availability, COVID-19 related closures). Spotting calls often began with setting an overdose response plan (i.e., when and who to call). Many participants noted that, due to the criminalization of drug use and fear of arrest, they preferred that roommates/friends/family members be called instead of emergency services in case of an overdose. Both spotters and spottees raised concerns about the timeliness of overdose response, particularly in remote and rural settings.

**Conclusion:**

Spotting is a novel addition to, but not replacement for, existing harm reduction services. To optimize overdose/COVID-19/STBBI’s prevention services, additional supports (e.g., changes to Good Samaritan Laws) are needed. The criminalization of drug use may limit uptake of formal spotting services.

## Background

For decades, people who use drugs (PWUD) have developed ways to help each other in general and also when governmental agencies have failed to provide needed support. Needle and syringe programs (NSP), overdose prevention sites (OPS), and supervised consumption sites (SCS) are examples of community-created and led initiatives designed to prevent or reduce drug-related morbidity and mortality among PWUD [1–3]. These programs were designed to modify the social, physical, economic, and political factors that interact to create a risk environment for PWUD [4, 5]. There has been a growing acknowledgement across drug policy and harm reduction research about the role risk environments play in perpetuating social and structural marginalization for PWUD and the need for interventions to operate at micro, meso, or macro levels [6] to reduce these harms [5, 6].

Spotting, a remote method of supervising drug consumption, represents a community driven intervention that allows for overdose response in environments where supervision of drug use is not available [7]. The practice of spotting is not new; for years some PWUD have contacted someone they trust to monitor their use of drugs using remote methods (by telephone, or more recently, using online video conferencing services) and intervene (either in person or by summoning emergency services) in case an overdose occurs. This remote method of supervision has become more salient during the lock-downs, stay at home orders, physical distancing requirements, service restrictions/closures and self-isolation orders during the COVID-19 pandemic. Indeed, formalized spotting services have begun to be implemented across Canada in the form of a call-centre and mobile apps [8, 9].

Access to supervision of consumption in person or by remote methods is important given the increases in overdose mortality observed since the beginning of the COVID-19 pandemic. Reports from the Government of Canada document that there were over 3,000 overdose related deaths across Canada from April to September 2020 as a result of the onset of the COVID-19 pandemic, which represented a 74% increase from the months of October 2019 to March 2020 [10]. Most overdose deaths are currently being driven by unregulated fentanyl and fentanyl analogues in the street-based opioid supply, with recent data indicating that fentanyl was a direct contributor to mortality in 87% of opioid-related deaths occurring during the COVID-19 pandemic in Ontario [11]. Similar reports have been documented by the Centre for Disease Control and Prevention, which state that over 80,000 overdose related deaths occurred across the United States from May 2019 to May 2020, with a substantial increase documented between the months of March and May 2020 as a result of the COVID-19 pandemic [12]. Additionally, unregulated benzodiazepines such as etizolam & flurazepam are being increasingly detected in fentanyl samples by drug-checking programs and during post-mortem toxicology, highlighting the volatility of the unregulated drug supply [13].

The increased rate of overdose deaths during COVID-19 follows several years of rapidly increasing overdose rates in Canada [10]. For example, Health Canada reported over 19,000 opioid-related deaths between January 2016 and September 2020 [10]. Canadian reports also indicate that 94% of overdose-related deaths were accidental and 80% of deaths involved fentanyl or fentanyl analogues in 2019 [14]. While harm reduction interventions such as SCS, OPS, NSP, and even a safe supply of drugs have expanded in number, PWUD continue to face access barriers (i.e., geographic accessibility, drug-related stigma, discrimination, or gender-related concerns such as violence and gendered stigmatization) [15–18]. Although the expansion of these harm reduction services have occurred in some provinces across Canada such as in Ontario, many others such as Newfoundland or Prince Edward Island, do not have or fund SCS or OPS spaces for these lifesaving services. Even within provinces which have implemented harm reduction services, there remains limited access to such services in urban, rural, and remote regions [19–21]. Lack of access has resulted in many PWUD using drugs alone in their private residences, increasing risk of overdose and drug-related morbidities [22–24]. For example, reports from Ontario, Canada indicate that three out of four overdose-related deaths which occurred during the COVID-19 pandemic took place when “no one was present to intervene” [11]. Literature from British Columbia, Canada document that PWUD engage with substance use alone as it improves convenience, comfort, and reduces experiences of stigma and discrimination [25].

Using data from an exploratory community-based research project, we aim to explore the ways in which spotting has been implemented *by and for* PWUD in two Canadian provinces- Nova Scotia and Ontario- during the COVID-19 pandemic and describe spotting motivations, processes, strengths, and limitations. Questions that were explored in this study and manuscript include how spotting practices operate, what the perceived benefits, challenges, and risks of spotting are, and for whom, and what the impact of spotting practices are for PWUD.

## Methods

For this exploratory qualitative study we recruited PWUD who had provided informal spotting services (spotters) and those who used informal spotting services (spottees). The study was approved by the University of Toronto Human Subjects Review Committee.

### Participant recruitment and sampling

Eligibility criteria for the study included: 1) having spotted and/or been spotted informally in the past 6 months; 2) being able to conduct the interview in English; 3) having access to a telephone or computer/internet; and 4) being willing to provide contact information (i.e., email address, full name, or postal address) to receive the honoraria by e-transfer, money gram, online gift card or by mail. Participants were recruited using personal and professional networks of study team members with lived/living expertise of drug use and through snowball sampling techniques. All members of the team have extensive experience in conducting harm reduction research and/or have lived/living experience of substance use and were consulted in the design of the study and study material. Given our recruitment strategy, certain participants had existing relationships with one of the interviewers. All participants were asked if they were comfortable with this prior to engaging with the study. Each potential participant was given the study phone number and email address. People who contacted the study team were screened for eligibility and were provided with an overview of the study objectives. We emailed a consent form to those who were interested and obtained verbal consent prior to participation. We recruited those who reported engaging in both formal (e.g., toll-free line operated by a harm reduction organization; n = 10) and informal (n = 20) spotting but this manuscript focuses exclusively on the latter group. The sample size for this study was determined a priori based on a recommended sample size [26]. We did indeed reach saturation of key themes (i.e., benefits, limitations, challenges) with this sample.

### Data collection methods

Interpretive descriptive [26] qualitative methods were used to conduct this study. All participants were asked to complete a brief socio-demographic questionnaire and semi-structured interview using a telephone. Participants were asked questions about: 1) how they learned about spotting – as a spotter and/or spottee and how spotting ‘works’; 2) what attracted them to use spotting either as a spotter or spottee; 3) by whom, where, and how often they engaged with spotting; 4) what platforms (e.g., telephone, FaceTime, and/or Zoom) were used to spot and how well these worked; and 5) what the perceived benefits (e.g., overdose, STBBI’s prevention, and/or COVID-19 prevention), challenges, and risks of spotting are for both spotters and spottees. If participants identified as both a spotter and spottee, they had the choice of what interview type (spotter vs spottee) they would like to complete. Each interview was conducted by two team members (MP, NK, CS, and/or GK), including one with lived experience of drug use, audio recorded, and uploaded onto an encrypted cloud. The interview guide was pilot tested with three participants and adapted according to their feedback. Interviews lasted between 30–60 min and were conducted between 08/2020–11/2020. All but one of the participants received a $30 honorarium by e-transfer, with the one individual preferring to receive it in cash.

### Data management and analyses

Once completed, interviews were professionally transcribed, corrected for accuracy, and managed using NVivo 12. Interpretive descriptive analytic methods were used to analyze the data; these methods are designed to excavate, illuminate, and articulate patterns and commonalities in qualitative data and to generate knowledge that is practical [26]. To develop the codebook, MP, CS, NK, and MB reviewed multiple transcripts and discussed appropriate codes. This preliminary codebook and themes which emerged from data analysis were shared with the larger team for feedback and adapted accordingly. Coding was completed in an iterative manner by two team members (MP, CS, NK, and/or MB) with discrepancies being discussed and resolved by a third team member. Demographic data were entered into RedCap and analyzed using descriptive statistics with Excel. Some quotations were modified to ensure confidentiality.

## Results

### Socio-demographic data

We recruited 20 PWUDs who provided or used informal spotting, of whom 10 identified themselves as spotters and 10 identified themselves as spottees. On average, participants described having people spot for them 12 days in the past month and having been a spotter for 4 days in the past month.

The average age of participants was 37 years, 35% self-identified as cisgender men (n = 7), 60% as cisgender women (n = 12) and 5% as transgender or gender-diverse (n = 1). The majority (75%) of the sample resided in southern Ontario (N = 15) and the remainder from Halifax & Cape Breton (N = 5) on the east coast of Canada. When asked about their ethnicity and racial background, 25% identified as Indigenous (n = 5), 10% as African, Black, or Caribbean (n = 2) and/or 90% as Caucasian (n = 18).

Spotters and spottees described similar informal spotting processes (e.g., how and why spotting is done), benefits (e.g., improved safety), limitations (e.g., locality), and recommendations on improving spotting practices (e.g., developing spotting guidelines). Each of these themes are outlined below.

### Informal spotting processes

Spotting was described as a long-standing community practice. Participants explained that the community previously referred to spotting as informal witnessing, but the name spotting caught on during the COVID-19 pandemic. When asked about the technology used for spotting, most participants reported using phones, text messaging, or video calls and being at home while spotting or while being spotted. Participants described informal spotting processes as starting with reaching out to a friend, family member, colleague, or another person with lived/living expertise of drug use by text, phone, or video call and asking them to ‘spot’. One participant described spotting with someone in this way:

So it would just involve me calling somebody. You know, they pick up, let them know that I'm going to be using at that time. And then, yeah, and then I would use and then stay on the phone with them for five minutes, and yeah, and I would give them the explicit instructions that if I don't respond or if my response is essentially nothing, then, it's probably a good idea to go ahead and call an ambulance. (Spottee 28)

Spotting calls most often included discussion of an overdose plan before the spottee used drugs. Issues such as whom to call, when to call, and what the spottee might do to reduce harm should they overdose were discussed on the majority of calls. For example, a spotter told us:

So, what I did was, I called the girl over FaceTime. We came up with a plan; so she didn't want me to call 911 because she was a parent. So, she said if she would overdose, to let her mom know and to guide her mom and how to use the naloxone and what to do, in that moment, should it happen. (Spotter 1)

When asked, many spottees said that they preferred that a partner, family member, neighbour or other person in close proximity to them be called in the event of an overdose. Many did not want the spotter to call emergency services because of fears of police accompanying paramedics on overdose calls, or fears of arrest. As one spotter expressed:

And I know a lot [spottees] are scared to even call anyone for help, because they're scared of being criminalized; they might have a warrant; they've had a bad experience with the police. A lot of men that I deal with are Indigenous, and they're very criminalized. And they trust me not to call the cops. (Spotter 12)

Some spotters also mentioned that they recommended putting extra drugs out of sight, pets in a safe place and unlocking the door. Once ready to use, spotters noted that they often chatted while the spottee used to ‘monitor’ what was going on and stayed on the line for 5–15 min after they had used drugs to ensure safety.

Fear of overdose – stated succinctly by one participant who explained ‘*Ah, cause I don't want to die.”* (Spottee 9) and/or a desire to have someone to talk to while using were reasons for spotting. All participants had concerns surrounding the high rates of drug-related overdoses and the unsafe drug supply. Spotting was offered a way to reduce social isolation and overdose risk elevated when COVID restrictions led harm reduction facilities to reduce the availability of services. Participant’s echoed narratives from previous research [27] surrounding fear of stigma, criminalization, and policing surrounding in person harm reduction services, which also facilitated their engagement with spotting. They used spotting as a strategy to try and stay safe during these multi public health emergencies.

Spotting frequently occurred from home, as it offered spottees the privacy they needed to feel safe from stigmatization, discrimination, the fear of criminalization of drug use and for some women and gender-diverse people, harassment and violence they experienced at some harm reduction programs. As a spottee explained:

[Spotting] is something that's easily accessible to people for when they're using alone, because they're likely to use alone anyway. And at least if I know that, like, people I care about are using alone, I can be, like, 'Call me.' You know, like, there's at least, like, an alternative safety net that offers some kind of support, that's more so than just if you have nothing in place. (Spottee 11)

Spotting on the go from places such as public parks or in cars, which did not lend itself to the same quality of privacy or safety, was also mentioned. Spotters continued to spot in non-ideal circumstances because they cared about the person who was reaching out to them for support.

Some spottees also explained that they used spotting because they wanted the safety of supervision but either did not have access to an SCS or did not feel comfortable using their drug of choice within those spaces. As one spottee explained:

I like the ability to, like, it feels safe, and I get to use in a place that's comfortable for me. So, like, an overdose prevention site, is not somewhere safe for me to use, if I'm using a substance that causes psychosis in me. Which I rarely, don't use those, like, I don't use ah, stimulants for that reason. But, I wouldn't be able to use a stimulant in an overdose prevention site, because I would go into psychosis, and it would feel very uncomfortable and I would be embarrassed, versus being in the comfort of my own home, where I feel like I have some privacy. (Spottee 3)

### Benefits of spotting

Participants described the benefits of spotting as providing access to overdose response whenever and wherever they needed it. This was particularly beneficial for people who smoked drugs, as supervised inhalation and smoking facilities are extremely limited in Canada, with most sites only allowing injection of drugs [28–31]. One spottee explained:

Well, you can't smoke, drugs in an overdose prevention site. So that's not even an option. So, the type of, the method in which I use my drugs doesn't work in the current model of how consumption and treatment sites are regulated in Ontario (Spottee 3)

Many participants explained that spotting helped to fill in a gap in service availability. One spottee expressed their hesitations in using SCS:

A lot of people just don't feel comfortable, either it's far removed from their communities, and it's just not, like, the scene they're used to being in, or it's just like, so unusual for them to use in such a like, formal medical setting, or even in front of people at all. Some people aren't close to a site. Some people use during hours that sites aren't open. Some people use only when they're with people, and some people, I don't know, just like, feel intimidated by sites in general. And some places don't even have site, so, yeah (Spottee 11)

Participants also highlighted the role of spotting in providing a sense of connection with members of their community. During the COVID-19 pandemic, while trying to adhere to physical distancing expectations, this connection was extremely important for those who typically used and shared drugs together. For some participants, the opportunity to talk to someone during a spotting call and develop a connection afforded them an opportunity to open up about past traumas. The informal conversations during spotting calls helped both parties develop trust:

I like spotting because spotting can save lives. Um, it's almost like a relationship. You get back from it what you put into it. So, if you build the trust with, like, a core group of people, inform them and you're like, open, and you're actually, like, honestly open with them, about, like, trying to let them understand why you tick the way you do, you know what I mean? Cause then, they can understand like, why you're doing what you're doing. So, they can counteract in term of help, in the right way. (Spottee 5)

Spotters described benefits of spotting both for spottees and for themselves in explaining how spotting improved their sense of pride and accomplishment. One spotter expressed:

I think the benefits of spotting are that people are safe. And that's mainly for the person who's using. The spotters, I think they can, I think, they, like, there's something to be said for feeling like you're accomplishing something, and feeling like you're helping someone. And I think that's what the spotters can take away from it. (Spotter 1)

### Limits of spotting: fear of delays, police, and the neighbours

When asked about the limitations of informal spotting, many said that because spotting was done remotely/virtually and often at a geographic distance, they worried about the consequences of delayed responses to an overdose event. Both spottees and spotters were anxious about any delays in reaching a person should they experience an overdose. One spotter shared their anxiety:

I've experienced anxiety – I, I get, like, I get anxiety. I worry about the person. Like, I worry if what I can do is going to, if what I'm doing is actually going to help save them in time. Like, I worry that because I'm looking for all these signs, and I have to make a phone call, and then I have to wait – like I worry that the overdoses won't be attended to in time. And, I worry about negative impacts of overdose, like, later on for them. But it's just like the anxiety of having to be hyper aware of someone else's situation. Yeah, that's the biggest one for me is anxiety. (Spotter 1)

Participants also expressed concern about what might happen if emergency services (e.g., ambulance) were called and repercussions for spottees from the police who often attend ambulance calls, and also from neighbours:

But I just think that police can do what they want. They can decide that a personal amount is an amount for trafficking. They can find other reasons to arrest people or they can just threaten you with arrest and be generally aggressive and make it an unpleasant situation, regardless. (Spottee 11)

The fear of criminalization stemming from police response during an overdose call was uniformly expressed by all participants and has been documented in the literature. This issue was particularly salient for participants who experienced intersecting oppressions. For example, one spottee who identified as a trans person described the following:

Police, 911 showing up on the call. That's like, that's like, your worst nightmare, especially having prior experience with, like, police brutality and police being, like, physical assholes. That is, that's my worst fear. Because it, like, the Good Samaritan law is only, it's at the discretion of the officer. So, how do you know that the officer won't take one look at you and say 'All right. So you are a Black person. You are obviously, you're obviously someone who has heroin and we need to take you in.' even if it's like, a small amount... for, for small personal usage, but once again, it's at the discretion of the officer. So who really knows, especially in this political climate. And we know how anti Black police are, systemically. So that doesn't really leave a lot of confidence. (Spottee 16)

This fear of criminalization was also acknowledged by participants when describing their willingness to engage in formalized spotting services, which often did not provide spottees with an opportunity to choose response plans (i.e., automatically call emergency services). Among participants who were aware of formalized spotting services, many described this policy as incredibly limiting and that it would deter them from wanting to use these services or from working within them.

### Emotional burden of the overdose crisis

Despite priding themselves on spotting, and the sense of community it created, spotters explained how spotting had a significant toll on their mental health and well-being. Many described experiencing trauma related to the constant fear and multiple losses as a result of the ongoing overdose crisis in Canada. This emotional burden limited how often they were willing to spot for others:

Because I think the problem is that right now, everybody is so burned out, that like, you almost feel like you're overburdening another person who already has so much on their plate, right? And so I think that, I mean, like, I think that this is exactly the issue, is that we're all so tired of this. (Spotter 7)

Spotting offered a reprieve from the trauma they rexperienced as a result of overdoses amongst friends, family, and community members. Spotters explained that calling others to help instead of physically responding themselves to an overdose helped minimize the trauma of intervening:

To be honest, the thing I like about spotting the most is that I can help someone, but I don't have to be the one to respond physically to the overdose. I can like whoever, like, say the parent of the person or I can call 911 for them, whatever they want me to do. But I don't actually have to be the one going through the trauma and the actions of saving someone's life. (Spotter 1)

### Recommendations for improving spotting

A recognition of the emotional burden that can accompany spotting underlies a recommendation to ensure spotters have access to supports – including debriefing supports – when necessary:

For people who are doing it informally, it would be nice if there was, like an awareness about a way to get support about, like, best practices or who to talk to about debriefing if something goes wrong. Cause like, nobody else knew that I was spotting in the scenario. So if he did, like, die for example, how do I go about talking about that, with other people […] So even just the place to for advice or debrief, and then, I could see how if one was offering that as part of like, a program, organized program, hopefully, those supports would be there, to some degree or another but offering it informally, I don't even know where I would necessarily, like, turn. (Spotter 30)

Other recommendations included compensation for spotters, guidelines for proper spotting practices, and improving accessibility, and awareness of spotting services among community members. As one spotter explained:

Like, so if there was funds available for these services, for people to have paid roles in this, so that we could not formalize something, cause I hate that word. […]But, I do like the sound of having funding for people to be paid for this service, so that it can be readily available when the user needs it, and um, so that it's not reliant on people just volunteering their time or you know, cause like, what if somebody needs me and I'm at my sister's wedding and nobody could pick up that time? (Spotter 6)

## Discussion

This study explored how informal spotting operated in the context of the COVID-19 pandemic and identified key benefits, concerns, and recommendations for the improvement of spotting. This is the first study to report on spotting in the literature and to address this issue during the COVID-19 pandemic. PWUD use spotting as a method to ensure that overdose response will be available if needed. They also highlighted that barriers to accessing appropriate harm reduction services including NSP/SCS and STBBI’s prevention services, as well as fears of criminalization made spotting an appealing ‘safety net’ in the context of a continuing overdose crisis. Among those who did not inject drugs, spotting provided access to overdose prevention which was otherwise inaccessible. Spotting has the potential to mitigate risk environments which are prevalent for PWUD and during the COVID-19 pandemic, has created opportunity for PWUD to connect with someone improving overdose prevention and physical distancing. Spotting’s role in facilitating overdose prevention during the pandemic is critical given closures and reduced capacity of in person harm reduction care [11, 24].

Even though spotting provides access in times and places where SCS are not available, the criminalization of drug use continues to act as a barrier to accessing this overdose prevention practice. As we document, spotting provided a perceived reprieve from the risk of criminalization faced when engaging with in person harm reduction services and when using in spaces other than one’s home. However, many feared arrest if 911 were called. This fear has been documented by others. Kolla and Strike [32] document fear of police amongst people operating SCS in their homes. Latimore and Bergstein [33] reported a similar concern in a study of PWUD in Baltimore, Maryland. The majority of participants feared arrest after calling emergency services for an overdose event. Many were not aware of the Good Samaritan Law which was designed to extend “immunity from low-level charges and/or parole violations to overdose victims or bystanders who call 911, or otherwise seek medical attention” [33, p. 3, 34]. Evans and colleagues [35] further substantiated these findings as they reported that among 198 people who used non-medical prescription opioids only 45.5% were aware of the Good Samaritan Law enacted in Rhode Island.

Changes to the Good Samaritan Law, policing practices, and emergency response policies are needed to ensure that PWUD can access services designed for them without fear of repercussion. Moallef and Hayashi [36] outline how knowledge of Good Samaritan Laws among police officers and emergency service responders is generally low across North America. Limited knowledge of this law amongst first responders facilitates negative experiences associated with overdose events for PWUD. Improving knowledge among these groups may therefore play a role in increasing the effectiveness of Good Samaritan Laws [36, 37]. Jakubowski and colleagues [38] reiterate this point by advocating for incorporating Good Samaritan Law knowledge within overdose prevention training programs and services which has the potential to improve the effectiveness of Good Samaritan Laws. However, increasing opportunities for knowledge dissemination surrounding Good Samaritan Laws do not address longstanding mistreatment of PWUD by the police. Scholars have recently called for the implementation of police non-attendance policies to overdose events [33, 36, 39]. Such policies have been implemented in Vancouver, Canada where Police Department members do not attend overdose events unless requested by emergency services [36, 39, 40]. The lack of attendance of police officers will minimize risk of harm experienced by PWUD and may improve the effectiveness of Good Samaritan Laws. While better training and changes in policies may alleviate some of the harms of criminalization, decriminalization of simple possession is what is needed [41].

These changes will be important in general and also in relation to other forms of spotting that exist in Canada including apps [8], peer witnessing [42], and several formal spotting telephone lines [9, 43]. Informal or formal spotting are tools for PWUD in this longstanding overdose crisis to reduce the number of people who use alone. Some research suggests that mobile app based technology in British Columbia may be beneficial in not only overdose prevention but in preventing co-occurring issues such as gender-based violence [44]. While our participants agreed that improved access to overdose response was crucial for their survival, they expressed hesitation to use these services if calling 911 was the policy in the event of an overdose. In addition, mobile apps come with distinct limitations such as accessibility concerns and general technology barriers [45].

Our study shares the limitations common to other qualitative studies related to size of the sample and transferability of findings. However, our exploratory community-based study benefited from the knowledge and connections of community members to rapidly recruit PWUD who may be difficult to recruit in general and in particular during a pandemic. As well, Damon et al. [46] outline the importance of using community based research approaches to minimize experiences of exploitation and stigma among PWUD.

## Conclusion

Spotting is a novel addition to, but not replacement for, existing harm reduction services. Spotting is useful in any setting but may be particularly useful in contexts without access to SCS and other harm reduction services. While formal overdose lines, apps, and SCS provide important options to reduce overdose risks, our participants expressed a desire to continue to connect informally to their friends and family for spotting. To improve spotting, guidelines which provide information on how to effectively spot (i.e., what questions to ask when spotting) and include safety information for spotters and spottees should be developed in partnership with community members engaging in spotting and PWUD [47]. More research is needed comparing informal, formal, and mobile app based spotting and each methods relative benefits and limitations as well as how spotting practices can improve response times in overdose event situations. Increasing awareness of spotting and support for those who engage with spotting holds potential for providing an additional safety net for PWUD during the overdose crisis.

## Data Availability

Data sharing is not applicable to this article.
